# The Aberdeen Weight-Bearing Test (Knee): a new objective test for anterior knee discomfort

**DOI:** 10.1007/s00068-018-0986-8

**Published:** 2018-07-20

**Authors:** David Robert Walker MacDonald, Haroon Rehman, Carol Ann Carnegie, Jordi Tomas-Hernandez, Alan John Johnstone

**Affiliations:** 1 Aberdeen Royal Infirmary, Foresterhill, Aberdeen, AB25 2ZN UK; 2 Vall d’Hebron Hospital, Barcelona, Spain

**Keywords:** Outcome measure, Clinical assessment, Knee, Anterior knee pain, Tibial nail

## Abstract

**Purpose:**

We present the Aberdeen Weight-Bearing Test (Knee), an objective test specific for anterior knee discomfort assessed via load bearing. We assess its validity by performing it on normal subjects with no knee symptoms and subjects who had undergone anterograde tibial nailing.

**Methods:**

Two scales are placed parallel on the floor with the dials concealed from the subject. The subject then kneels with one knee on each scale. The weight through each knee is recorded at 0, 15, 30, 45, and 60 s. The proportion of total body weight on each leg at each timepoint is calculated, and a ratio calculated from the values. A value of 1 equates to equal weight on each leg. The test was performed on 53 normal subjects and 38 subjects who had undergone tibial nailing.

**Results:**

In the normal group, no significant difference in mean ratio of weight distribution (left:right) was seen at any timepoint (mean ratio range = 0.98–0.99, *p* value range = 0.18–0.64). In the tibial nail group, a difference was observed in mean ratio of weight distribution (injured:uninjured) favouring the uninjured leg, reaching significance at 0, 15, 30, and 45 s (mean ratio range = 0.88–0.94, *p* value range = 0.01–0.02). At 60 s, the mean ratio was 0.93 (*p* = 0.09).

**Conclusion:**

The Aberdeen Weight-Bearing Test (Knee) is an objective, easily reproducible, specific test for anterior knee discomfort. It produces different results in individuals who have undergone anterograde tibial nailing compared to individuals with no knee symptoms.

## Introduction

Anterior knee discomfort following anterograde intramedullary nailing of the tibia is a widely recognised complication which can cause significant morbidity and disability [1]. It occurs commonly with incidences between 8.7 and 37% reported [2, 3]. There is increasing interest in the literature in the use of the suprapatellar approach rather than the traditional infrapatellar approach as this may reduce this complication. Although to our knowledge, no randomised controlled trial has been published, other studies comparing these approaches have used subjective patient-reported outcome measures (PROMs) rather than objective outcome measures [4, 5].

The PROMs used such as the Oxford knee score and Lysholm knee score are not specific for anterior knee pain, but rather reflect overall knee function [6, 7]. Whilst PROMs such as the Kujala score [8] and Fulkerson’s modification of the Lysholm score [9] are more specific for anterior knee pain, they suffer from the same disadvantages as other PROMs.

PROMs are frequently used as outcome measures as they reflect a patient’s self-perceived burden of disease, which is important in clinical practice. They are readily available and can be posted to patients or completed online. However, they have significant drawbacks. These include “floor” and “ceiling” effects [10] such as the preoperative floor effects and postoperative ceiling effects seen in the WOMAC and SF-36 scores of patients undergoing total hip arthroplasty [11], and the ceiling effects seen in the Harris hip scores of patients undergoing total hip arthroplasty [12]. PROMs can also give mixed messages and can be negatively influenced by psychosocial factors [13, 14], measuring the patient’s perception of an outcome, rather than the true outcome.

To adequately assess the impact of the suprapatellar approach compared to the infrapatellar approach on anterior knee pain, it is necessary to use objective outcome measures in addition to PROMs. Detailed measurement of joint motion and muscle activity using equipment such as pedometers and accelerometers can be a useful objective outcome measure. However, this requires specialist equipment and training only available in certain centres. These problems can be addressed using objective performance-based functional outcome tools in which the patient is observed performing tasks such as walking, rising from a chair or climbing stairs, and their performance quantified in time, counting or distance [15]. Examples include the Timed Up and Go Test [16], 6-Minute Walk Test [17] and Stair Climbing Test [18]. These do not correlate well with PROMs suggesting that PROMs may not fully reflect functional performance [15]. Functional outcome tools are often also not specific to site and pathology.

We present The Aberdeen Weight-Bearing Test (Knee) (AWT-K), an objective test specific for anterior knee discomfort assessed via direct load bearing. We assessed its validity by performing it on subjects with no knee symptoms and subjects who had undergone anterograde tibial nailing. It may be a useful objective outcome measure to quantify anterior knee discomfort following anterograde tibial nailing.

## Methods

### Aberdeen Weight-Bearing Test (Knee)

**Fig. 1:**
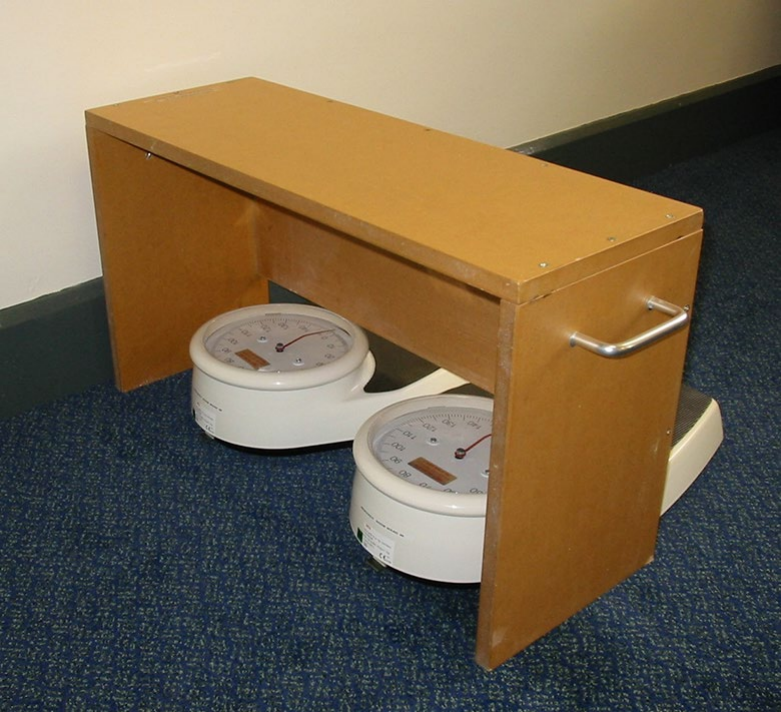
Two scales are placed on the floor with dials concealed from the subject

**Fig. 2:**
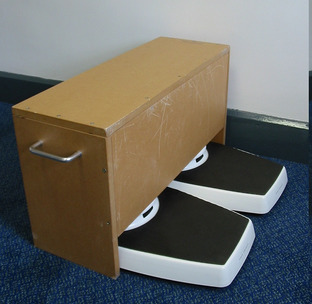
Two scales are placed on the floor with dials concealed from the subject

Two equally calibrated scales are placed in a parallel configuration on the floor. The dials are concealed from the test subject by a box (Figs. 1, 2).

**Fig. 3:**
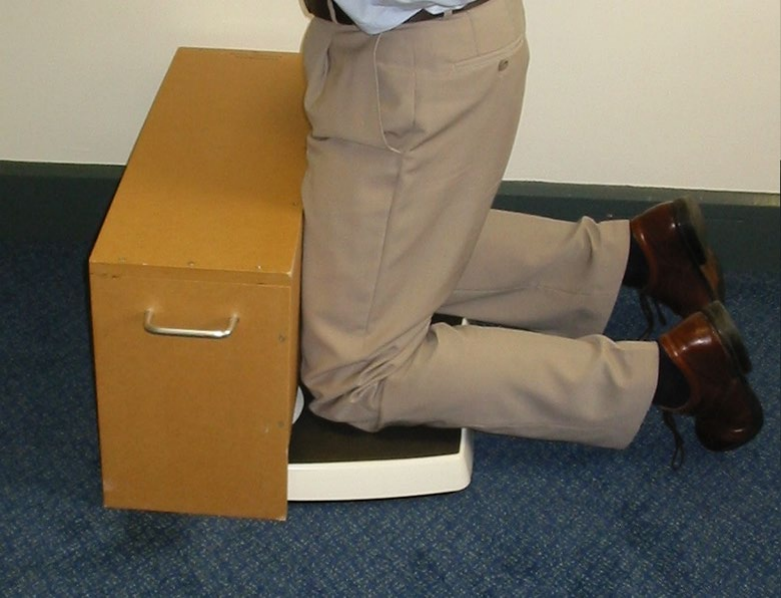
Subject kneels with one knee on each scale

Patient weight is calculated as the sum of the scale readings as the subject stands with one foot on each scale. The subject then kneels with one knee on each scale. The anterior thighs should be gently pressed against the box to maximise weight through the knee (Fig. 3). The feet may touch the ground behind the scales to maintain balance, but should bear minimum weight.

The weight passing through each knee is then recorded at 0, 15, 30, 45, and 60 s. The test ends at 60 s, or if the subject is unable to continue the test for the full 60 s.

To account for differences in total weight between subjects, the weight on each knee is converted to a proportion of the subject’s total body weight. A ratio is calculated from the values (injured knee weight/total weight on scales):(uninjured knee weight/total weight on scales), with a value of 1 representing equal weight on each knee. In our study in subjects who had sustained an injury, the injured leg was compared to the uninjured leg. In uninjured patients, the left leg was compared to the right.

### Study design

**Fig. 4:**
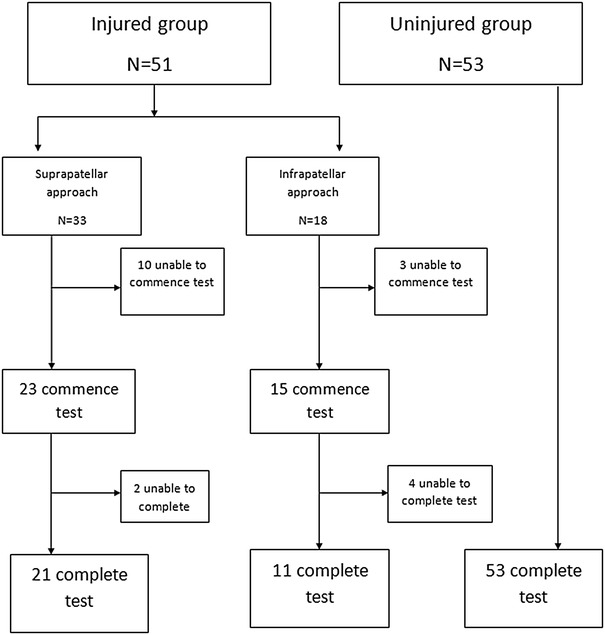
Injured and uninjured groups

To determine whether the Aberdeen Weight-Bearing Test (Knee) produced different results in subjects with lower limb injury and uninjured subjects, the test was performed on 104 individuals. The normal population was represented by 53 subjects with no history lower limb injury or pathology. The injured population was represented by 51 subjects who had undergone anterograde intramedullary tibial nailing using either a suprapatellar (33) or infrapatellar (18) approach (Fig. 4). The test was performed at a mean of 27 months after surgery (range 6–52 months, SD = 14). The individuals also completed Fulkerson [9] and Irrgang’s scores [19] (commonly used PROMs assessing knee function) at the same review point and these were correlated with their weight-bearing test results. Prior to data collection, our scales were calibrated using standard 10 kg weights.

The Statistical Package for the Social Science SPSS v20 (SPSS Inc, Chicago, IL, USA) was used to analyse the data. A one-tail *t* test with a test value of 0 was used to assess weight differences between limbs in injured and uninjured patients. A one-tail *t* test with a test value of 1 was used to assess ratio of weight distribution in injured and uninjured patients. The spearman’s rank correlation test was used to assess the relationship between AWT-K, Fulkerson, and Irrgang scores.

## Results

A total of 104 subjects were recruited. The normal population was represented by 53 subjects. 51 patients that had undergone intramedullary nail fixation for tibial shaft fractures were tested. A suprapatellar approach was used in 33 patients and an infrapatellar approach in 18 patients. Of the 51 injured patients, 13 were unable to commence the kneeling test due to pain and were excluded. Of the remaining 38, 6 were unable to complete the test due to pain (Fig. 4). Demographic data is provided in Table 1.

**Table 1:** Demographic data for injured and uninjured groups

	Sample size	Age	Male	Female	Weight
Normal	53	42 ± 13 (22–60)	26 (49%)	27 (51%)	77.6 ± 15.4 (48–120)
Injured	51	42 ± 13 (22–68)	37 (73%)	14 (27%)	84.1 ± 18.7 (60–170)

### Normal population

No significant difference in weight distribution (kg) across the knees was detectable for our test population at any time interval (Table 2). Similarly, there was no significant difference in the ratio of weight distributed through both limbs (left:right) (Table 3).

**Table 2:** Mean difference in weight distribution for normal group

	*N*	Mean difference weight distribution left–right (kg)	SD	Sig.
0 s	53	− 0.1	3.9	0.33
15 s	53	− 0.9	4.0	0.11
30 s	53	− 0.9	4.0	0.11
45 s	53	0	3.8	0.37
60 s	53	− 1.1	4.3	0.08

**Table 3:** Mean ratio of weight distribution for normal group

	*N*	Mean ratio weight distribution left:right	SD	Sig.
0 s	53	0.99	0.11	0.64
15 s	53	0.98	0.11	0.22
30 s	53	0.98	0.11	0.21
45 s	53	0.99	0.11	0.62
60 s	53	0.98	0.12	0.18

### Injured population

A significant difference in weight distributed across the knees was observed at all timepoints (Table 4). A significant difference in the ratio of weight distributed across each knee (as a proportion of total weight) was observed at 0, 15, 30, and 45 s, and there was greater variability in the ratio of weight tolerated through injured limbs (Table 5).

**Table 4:** Mean difference in weight distribution for injured group

	*N*	Mean difference weight distribution injured–uninjured	SD	Sig.
0 s	38	4.7	11.7	0.03
15 s	37	5.5	9.1	0.001
30 s	36	4.0	9.3	0.01
45 s	35	4.6	9.4	0.01
60 s	32	3.1	8.4	0.05

**Table 5:** Mean ratio of weight distribution for injured group

	*N*	Mean ratio weight distribution injured:uninjured	SD	Sig.
0 s	38	0.91	0.25	0.03
15 s	37	0.88	0.22	0.002
30 s	36	0.92	0.23	0.04
45 s	35	0.91	0.22	0.02
60 s	32	0.94	0.20	0.09

Although there was a trend toward decreasing correlation with the Fulkerson score as time progressed, no statistical significance was noted. A statistically significant correlation was observed between the AWT-K at 45 s and the Irrgang score (*p* < 0.05) (Table 6). A statistically significant correlation was noted between the Irrgang and Fulkerson scores.

**Table 6:** Correlation between AWT-K, Fulkerson, and Irrgang scores

	Spearman’s correlation coefficient	
	Fulkerson score	Irrgang score
AWT-K 0 s *N* = 38	0.418 *p* = 0.01	0.247 *p* = 0.14
AWT-K 15 s *N* = 37	0.326 *p* = 0.05	0.157 *p* = 0.36
AWT-K 30 s *N* = 36	0.353 *p* = 0.04	0.147 *p* = 0.39
AWT-K 45 s *N* = 35	0.308 *p* = 0.07	0.089 *p* = 0.61
AWT-K 60 s *N* = 32	0.336 *p* = 0.06	0.196 *p* = 0.28
Fulkerson score *N* = 38	1.000	0.862 *p* < 0.001
Irrgang score *N* = 38	0.862 *p* < 0.001	1.000

## Discussion

We believe that the Aberdeen Weight-Bearing Test (Knee) is an easily reproducible outcome measure for anterior knee discomfort assessed via direct load tolerance. Unlike other outcome measures, it is objective, and specific to anterior knee discomfort. It does not require specialist equipment or training, and can be easily performed in an outpatient setting. Our results demonstrate that the test produces different results in individuals who have been treated using an anterograde tibial nail, compared to normal individuals.

The differences in AWT-K result were significant at all but one timepoint in injured patients. It is likely that significance was not reached at 60 s due to the limited number of injured subjects in our study. It seems likely that with further recruitment, the difference found will reach significance.

A potential weakness of the test is the possibility that a subject could unevenly shift his weight onto his feet during the test, altering the test result. However, our data show a consistency in mean proportion of total body weight on the knees compared to the feet across all timepoints in both groups. In the uninjured group, the mean proportion of total body weight through the scales compared to the feet ranged from 0.96 to 0.97, whilst in the injured group, it ranged from 0.90 to 0.91. Another potential weakness is that 13 of the 51 injured patients were unable to commence the weight-bearing test due to pain, and a further six patients were unable to complete the test due to pain. However, this itself can be used as part of the outcome measure, and is a useful indication of level of function. A further weakness of the study is that the AWT-K was performed at variable time period following surgery in the injured group, and this is likely to have influenced results. This does not alter our conclusion that the AWT-K produces different results in patients who have undergone anterograde tibial nailing compared to normal individuals, but if used in future studies the, the AWT-K will be more meaningful if used at regimented timepoints.

Our results show a poor correlation between the AWT-K and the subjective Fulkerson and Irrgang tests. The poor correlation may indicate a poor correlation between subjective patient-reported symptoms and the objective measurements, as has previously been shown [20]. It may also have been influenced by the heterogeneity of our injured sample group. Some had their surgery performed via an infrapatellar approach and some via a suprapatellar approach that may give a lower incidence of anterior knee discomfort. We plan to perform subgroup analysis to assess this possibly following further patient recruitment to this study.

Although PROMs have value in determining patients’ self-perceived burden of disease, they are affected by psychosocial factors and many are not specific. By contrast, the AWT-K is an objective outcome measure for anterior knee discomfort assessed through load bearing. We have demonstrated its use as an outcome measure for anterior knee discomfort following anterograde tibial nailing. In the future, it may be used as an outcome measure in other procedures or conditions known to produce anterior knee pain such as anterior cruciate ligament reconstruction or chondromalacia patellae. It may also be easily modified to be used as an outcome measure for other lower limb procedures or conditions.

## Conclusion

We present the Aberdeen Weight-Bearing Test (Knee); an objective test specific for anterior knee discomfort assessed via load bearing. We conclude that the AWT-K produces different results in individuals who have undergone anterograde tibial nailing compared to normal individuals. It may be a useful, objective outcome measure following anterograde tibial nailing, as well as other procedures and conditions.
